# The human limbus functions as an instructive epithelial transition zone

**DOI:** 10.21203/rs.3.rs-9948733/v1

**Published:** 2026-06-24

**Authors:** Yuzuru Sasamoto, Yoshiko Fukuda, Catherine Lee, Shuoshuo Wang, Sheethal Umesh, Shinri Sato, Kosei Suzuki, Shoko Kiritoshi, Ioannis Vlachos, Nick Di Girolamo, Markus Frank, Natasha Frank

**Affiliations:** Department of Ophthalmology, University of Washington; Department of Ophthalmology, Boston University Chobanian & Avedisian School of Medicine; Brigham and Women’s Hospital; Beth Israel Deaconess Medical Center; Spatial Transcriptomics Unit, Beth Israel Deaconess Medical Center; Department of Ophthalmology, University of Washington; University of Washington; Division of Genetics, Brigham and Women’s Hospital, Harvard Medical School, Boston; Cancer Research Institute / Harvard Medical School Initiative for RNA Medicine; University of New South Wales; Transplant Research Program, Boston Children’s Hospital, Harvard Medical School; Brigham and Women’s Hospital

## Abstract

The corneal and conjunctival epithelia have traditionally been regarded as distinct lineages separated by the limbus, a specialized niche proposed to enforce irreversible corneal identity through a hierarchically organized limbal stem cell compartment^1–3^. In this prevailing model, conjunctivalization of the cornea reflects failure of a lineage barrier. Here, we show that the adult human limbus does not function as a rigid boundary but rather as an instructive epithelial transition zone. Integrated single-cell transcriptomics, spatial mapping, and clonal functional analysis demonstrate that limbal basal progenitors are transcriptionally aligned with conjunctival basal cells and are intrinsically bipotent, capable of generating either conjunctival or corneal epithelium. Spatial profiling redefines the functional conjunctival-corneal boundary at the inner edge of the limbus and reveals a sharp transition between conjunctival-type and corneal-type basal programs. Mechanistically, we identify keratinocyte growth factor (KGF) derived from limbal fibroblasts as a niche signal that instructs corneal fate through enhanced PAX6 protein abundance and FGFR2-dependent activation of MYC, thereby coupling lineage specification with proliferative expansion. In contrast, epidermal growth factor (EGF) sustains conjunctival identity, establishing growth factor balance as a molecular switch governing epithelial fate at this interface. These findings redefine the human limbus as a dynamic fate-determining niche and demonstrate that epithelial identity in adult human tissue remains instructable.

## Introduction

Epithelial tissues are commonly organized into anatomically distinct compartments maintained by lineage-restricted stem cell populations. At transition zones between epithelia, these boundaries are often interpreted as sites of strict lineage segregation that preserve tissue identity. The human ocular surface has served as a canonical example of this paradigm: corneal and conjunctival epithelia are regarded as distinct lineages separated by the limbus, which is thought to enforce corneal identity through a hierarchically organized limbal stem cell compartment. In this prevailing model, centripetal migration of limbal progenitors sustains the corneal epithelium, and conjunctivalization of the cornea in limbal stem cell deficiency reflects failure of a lineage barrier. Whether the human limbus functions as a rigid boundary or a dynamic transition zone in which epithelial fate remains instructable remains unresolved. Here, we integrate single-cell transcriptomics, spatial profiling, and clonal functional assays across the adult human ocular surface to define epithelial organization at single-cell resolution. We demonstrate that basal progenitors at the limbus are transcriptionally aligned with conjunctival basal cells and intrinsically bipotent, capable of generating either conjunctival or corneal epithelium. Spatial mapping repositions the functional conjunctival-corneal boundary to the inner edge of the limbus and identifies a sharp transition between basal programs. Mechanistically, we show that KGF derived from limbal fibroblasts instructs corneal identity by enhancing PAX6 protein abundance and FGFR2-dependent activation of MYC, whereas EGF instructs conjunctival identity, thereby establishing growth factor balance as a molecular switch governing epithelial fate at this interface.

These findings redefine the limbus as an instructive epithelial transition zone and provide a framework in which adult epithelial identity is actively programmed by niche-derived signals rather than irreversibly pre-committed.

### Cellular and spatial mapping redefine the corneal-conjunctival boundary

**Figure 1:**
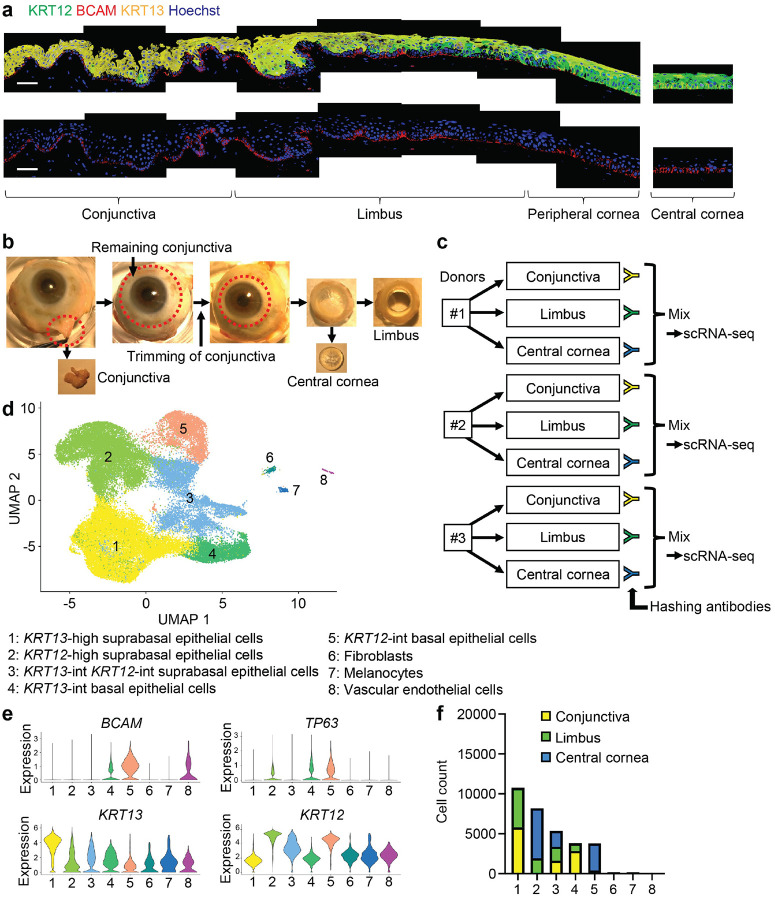
Heterogeneity of the human ocular surface epithelium. **a**, Representative immunofluorescence staining of KRT12 (green), BCAM (red), and KRT13 (yellow) across the human ocular surface, spanning conjunctiva, limbus, peripheral cornea, and central cornea. Nuclei stained with Hoechst 33342 (blue). n = 3 donors. Scale bar: 50μm. **b**, Stepwise dissection of the human eye globe illustrating isolation of conjunctiva, limbus, and central cornea. See Methods section for details. **c**, Schematic overview of sample preparations for CITE-seq. Cells isolated from the conjunctiva, limbus, and central cornea of three donors were labeled with cell hashing antibodies prior to scRNA-seq. **d**, UMAP of 45,042 cells from three human donors. Clusters 1–8 were annotated based on characteristic gene expression signatures. int, intermediate. **e**, Violin plots showing expression of *BCAM*, *TP63*, *KRT13*, and *KRT12* across identified clusters in CITE-seq. **f**, Distribution of cells by anatomical origin across clusters.

The phenotypic boundary between the corneal and conjunctival epithelia has long been presumed to reside at the outer edge of the limbus^9^. To directly interrogate this anatomical model, we performed comprehensive immunohistochemical mapping across the full human ocular surface. The conjunctival epithelial marker KRT13^10^ extended throughout the limbal region and reached the inner edge of the limbus, where its expression overlapped with the corneal epithelial marker KRT12^7,11–13^ (Fig. 1a). Most suprabasal epithelial cells in both conjunctiva and limbus expressed KRT13, whereas KRT12 was confined to suprabasal limbal cells and to all epithelial layers of the peripheral and central cornea, and was absent from conjunctiva (Fig. 1a).

We next examined BCAM, a recently identified marker of ocular surface progenitor cells^14–16^. BCAM was continuously expressed in basal epithelial cells across the conjunctiva, limbus, and cornea. However, BCAM-positive basal cells in the conjunctiva and limbus did not express the differentiation markers KRT13 or KRT12, whereas BCAM-positive basal cells in the peripheral and central cornea co-expressed KRT12, consistent with prior observations^16^. These findings reposition the functional corneal-conjunctival boundary to the inner edge of the limbus. This interface is demarcated by BCAM-positive KRT12/KRT13-negative basal epithelial cells on the conjunctival side and BCAM/KRT12-positive basal epithelial cells on the corneal side (Fig. 1a). The coexistence of KRT12- and KRT13-positive suprabasal cells within the limbus further supports the presence of progenitors capable of contributing to both epithelial lineages.

### Single-cell transcriptomics reveals transitional basal states at the limbal interface

To resolve limbal epithelial heterogeneity at single-cell resolution, we performed single-cell RNA sequencing (scRNA-seq) coupled with cell hashing (CITE-seq)^17,18^. Epithelial cells were independently isolated from conjunctiva, limbus, and central cornea of three human donors (n = 3; Fig. 1b), labeled with sample-specific hashing antibodies, pooled, and subjected to droplet-based scRNA-seq (Fig. 1c). Dimensionality reduction using Uniform Manifold Approximation and Projection (UMAP)^19^ resolved eight transcriptionally distinct clusters (Fig. 1d; **Extended Data** Fig. 1a).

Cluster identities were assigned based on canonical marker expression (Fig. 1e; **Extended Data** Figs. 1b, c; **Extended Data Table 1**). High *BCAM* and *TP63* expression defined basal epithelial progenitor cells, whereas lower expression marked suprabasal populations. Among differentiated suprabasal cells, cluster 1 was enriched for *KRT13*-high cells, cluster 2 for *KRT12*-high cells, and cluster 3 comprised cells co-expressing *KRT13* and *KRT12*. Basal progenitors segregated into cluster 4 (*KRT13*-intermediate) and cluster 5 (*KRT12*-intermediate). Non-epithelial populations, including *FBLN1*-positive fibroblasts (cluster 6), *MLANA*-positive melanocytes (cluster 7), and *PECAM1*-positive vascular endothelial cells (cluster 8), were also identified (**Extended Data** Fig. 1c; **Extended Data Table 1**). Hashing-based cell-of-origin analysis demonstrated region-specific contributions to clusters (Fig. 1f). Clusters 1 and 4 were derived predominantly from conjunctiva and limbus, whereas cluster 2 originated primarily from limbus and central cornea. Cluster 3 contained cells from all three anatomical regions. In contrast, cluster 5 was composed largely of central corneal cells (Fig. 1f).

Notably, limbus-derived *BCAM*-positive/*TP63*-positive basal epithelial cells were distributed across clusters 4 and 5. Importantly, 72.7% of them co-clustered with conjunctival basal epithelial cells (cluster 4), rather than corneal basal epithelial cells (cluster 5), indicating that limbal basal cells are transcriptionally more similar to conjunctival basal cells than to central corneal basal cells. This pattern is consistent with immunohistochemical findings showing a comparatively less differentiated phenotype in limbal basal epithelium (Fig. 1a). Differential expression analysis within cluster 4 revealed only three genes, *MT2A*, *CXCL14*, and *ID1*, that distinguished limbus- from conjunctiva-derived cells (log2 fold change > |2|, p < 10^−^^6^) (**Extended Data** Fig. 1d), underscoring the high transcriptional concordance between these compartments. Lineage trajectory inference using Slingshot^20^ indicated that conjunctival and corneal basal epithelial clusters (clusters 4 and 5) were not directly connected but instead converged through cluster 3 (**Extended Data** Fig. 1e), consistent with the presence of a transitional intermediate state. Furthermore, recently described quiescent limbal stem cell markers^21,22^ were heterogeneously distributed within the basal compartment: *GPHA2* and *IFITM3* were enriched in subsets of cluster 4, and *CD63*^21^ was detected across multiple epithelial clusters (**Extended Data** Fig. 1f). Together, these single-cell analyses suggested that the limbal basal epithelium occupies a transitional position between conjunctival and corneal programs.

### Spatial transcriptomics resolves the basal epithelial interface in situ

**Figure 2:**
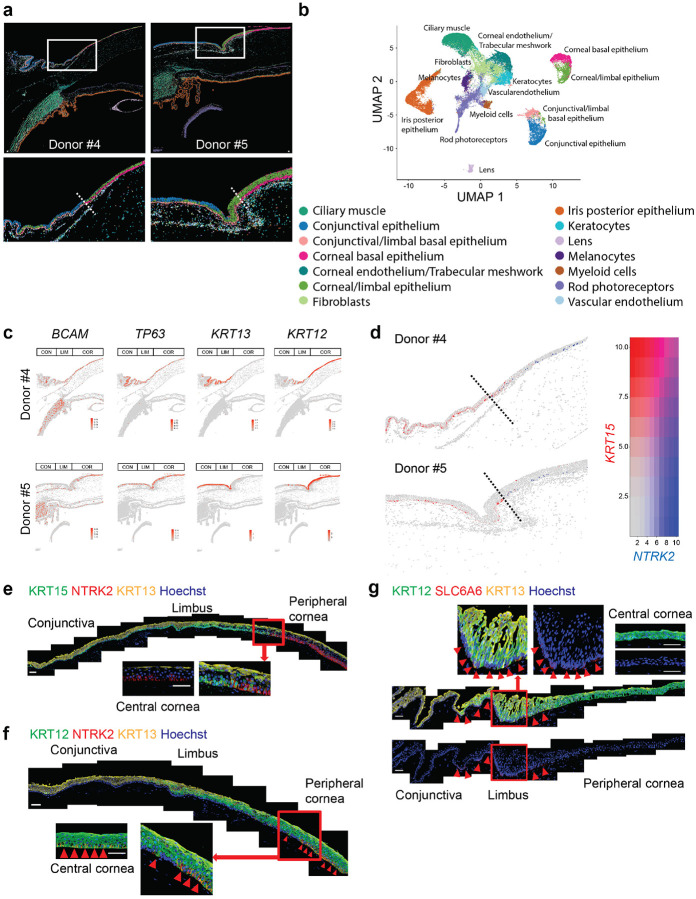
Phenotypic transition of basal epithelial cells across the human ocular surface. **a,** Spatial annotated maps of corneal sections from two human donors (Donors #4 and #5) generated using Visium HD. Lower panels show higher-magnification views of the boxed regions in the upper panels. The dotted lines delineate the inner boundary of the limbus. **b,**UMAP representation of spatial transcriptomic profiles. Cluster identities were assigned on the basis of characteristic gene expression signatures (**Extended Data Fig. 2b**). **c,** Spatial feature plots depicting the distribution and relative expression levels of *BCAM*, *TP63*, *KRT13*and *KRT12* across conjunctiva, limbus and cornea. CON, conjunctiva; LIM, limbus; COR, cornea. **d,** Spatial co-expression maps of *KRT15*and *NTRK2*. The dotted lines delineate the inner boundary of the limbus. **e,** Representative multiplex immunofluorescence staining of KRT15 (green), NTRK2 (red), and KRT13 (yellow) across the ocular surface, spanning conjunctiva, limbus, peripheral cornea to central cornea. Nuclei are counterstained with Hoechst 33342 (blue). n = 3 donors. Scale bar: 50μm. **f,** Representative multiplex immunofluorescence staining of KRT12 (green), NTRK2 (red), and KRT13 (yellow) across the ocular surface regions indicated. Arrowheads denote NTRK2-positive basal epithelial cells. Nuclei are counterstained with Hoechst 33342 (blue). n = 3 donors. Scale bar: 50μm. **g,** Representative multiplex immunofluorescence staining of KRT12 (green), SLC6A6 (red), and KRT13 (yellow) across the ocular surface. Arrowheads indicate SLC6A6-positive basal epithelial cells. Nuclei are counterstained with Hoechst 33342 (blue). n = 3 donors. Scale bar: 50μm.

To directly map this interface in tissue context, we performed 10x Visium HD spatial transcriptomics on two donor corneas (Figs. 2a, b; **Extended Data** Figs. 2a, b). Spatial profiles recapitulated the epithelial subpopulations defined by CITE-seq, confirming reproducibility across platforms. The ocular surface segregated into spatially distinct subpopulations: KRT13-positive suprabasal conjunctival epithelium, *KRT12*-positive suprabasal corneal/limbal epithelium, *BCAM/TP63*-positive *KRT13*-weak-positive conjunctival/limbal basal epithelial cells, and *BCAM/TP63/KRT12*-positive corneal basal epithelial cells (Figs. 2a–c). In both datasets, the transition between basal epithelial identities localized precisely to the inner edge of the limbus (Fig. 2a). In CITE-seq (**Extended Data** Fig. 1g), *KRT15* and *SLC6A6* were enriched in conjunctival basal epithelial cluster 4, whereas *NTRK2* was enriched in corneal basal epithelial cluster 5. Spatial mapping confirmed an interface between *KRT15*-positive conjunctival basal cells and *NTRK2*-positive corneal basal cells at the inner limbal margin (Fig. 2d; **Extended Data** Fig. 2c).

Immunostaining validated this boundary architecture. KRT15 expression was confined to basal epithelial cells of conjunctiva and limbus, with additional labeling in a subset of limbal suprabasal cells (Fig. 2e). In contrast, NTRK2 co-localized with KRT12 and was restricted to the basal epithelial layer beginning at the inner limbal border and extending centrally into the cornea (Fig. 2f). The concordance across CITE-seq, spatial transcriptomics, and protein-level validation refines the conjunctival-corneal basal epithelial boundary to the inner edge of the limbus.

Notably, although *SLC6A6* RNA expression did not differ significantly between limbus and conjunctiva within cluster 4, SLC6A6 protein was elevated in limbal basal epithelial cells relative to conjunctival basal cells (Fig. 2g; **Extended Data** Fig. 1h), suggesting post-transcriptional or post-translational regulation at this transitional interface.

### KGF-FGFR2 signaling instructs corneal lineage specification in bipotent basal progenitors

CITE-seq and spatial transcriptomics revealed striking phenotypic concordance between limbal and conjunctival basal epithelial cells (Figs. 1f, 2a, and 2b), while *KRT12*-positive corneal epithelial cells were detected within the suprabasal limbal compartment (Figs. 1a and 2c), positioning corneal and conjunctival programs in close spatial proximity. These observations suggested that limbal basal epithelial cells may remain bipotent and responsive to local instructive cues.

**Figure 3:**
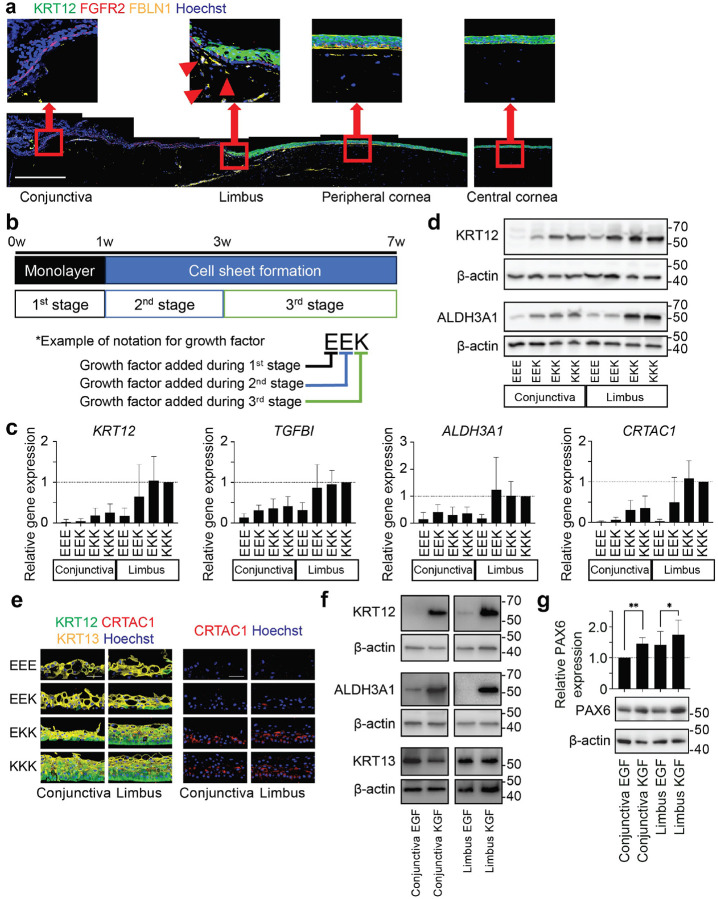
KGF drives phenotypic conversion of conjunctival epithelial cells. **a**, Representative immunofluorescence staining of human ocular surface tissue spanning conjunctiva, limbus, peripheral cornea and central cornea, showing KRT12 (green), FGFR2 (red), and FBLN1 (yellow). Nuclei are counterstained with Hoechst 33342 (blue). Arrowheads indicate FBLN1-positive cells. n = 3 donors. Scale bar: 50μm. **b**, Schematic of the stratification protocol. Culture was divided into three stages: pre-stratification (0–1 weeks), the first two weeks of stratification (1–3 weeks), and the last four weeks of stratification (3–7 weeks). w, weeks; E, EGF; K, KGF. **c**, Relative mRNA expression of *KRT12*, *TGFBI*, *ALDH3A1* and *CRTAC1*under the indicated culture conditions. Data are presented as mean ± SD. n = 7 donors. **d**, Representative immunoblot analysis of KRT12 and ALDH3A1 under the indicated cell culture conditions. β-actin serves as a loading control. n = 4 donors. **e**, Representative immunofluorescence staining of KRT12 (green), CRTAC1 (red), and KRT13 (yellow) in conjunctival- and limbal-derived epithelial cell sheets cultured under the indicated conditions. Nuclei are counterstained with Hoechst 33342 (blue). Right panels show CRTAC1 and Hoechst 33342 images extracted from the left panels. n = 3 donors. Scale bar: 50μm. **f**, Representative immunoblot analysis of KRT12, ALDH3A1, and KRT13 in cell sheets derived from a single BCAM-positive conjunctiva or limbal epithelial cell. β-actin serves as a loading control. n= 3 donors. **g,** Top, quantification of relative PAX6 protein expression normalized to β-actin in monolayer conjunctival and limbal epithelial cells cultured with EGF or KGF (mean ± SD). Bottom, representative immunoblot of PAX6. n = 5 donors. *p < 0.05, and **p < 0.01 by paired t-test.

KGF (also known as FGF7), secreted by limbal fibroblasts, has been implicated in corneal epithelial differentiation^23–25^. Consistent with this paradigm, CITE-seq and spatial profiling identified robust *FGF7* expression in *FBLN1*-positive limbal fibroblasts (cluster 6; **Extended Data** Fig. 3a). Immunostaining demonstrated expression of FGFR2, the high-affinity KGF receptor^26,27^, across basal epithelial compartments from conjunctiva to central cornea (**Extended Data** Fig. 3b), with FBLN1-positive fibroblasts positioned adjacent to FGFR2-positive limbal basal epithelial cells (Fig. 3a). These findings establish the anatomical framework for paracrine KGF-FGFR2 signaling at the limbal interface.

To determine whether KGF is sufficient to direct corneal lineage specification, primary human conjunctival and limbal epithelial cells were cultured under defined growth factor conditions (Fig. 3b). During initial expansion, the majority of conjunctival (94.9 ± 4.5%, n = 4) and limbal (97.1 ± 2.8%, n = 3) epithelial cells expressed the basal progenitor marker BCAM^16^ (**Extended Data** Fig. 3c), indicating preservation of progenitor identity. Upon stratification, KGF exposure induced corneal differentiation in both conjunctival- and limbal-derived epithelial sheets, characterized by upregulation of KRT12, TGFBI, ALDH3A1, and CRTAC1^13,28–31^ (Fig. 3c; **Extended Data Table 2**), with robust protein induction confirmed by immunoblotting and immunostaining (Figs. 3d, 3e). CRTAC1 co-localized with KRT12 in differentiated limbal and corneal epithelia *in vivo* (**Extended Data** Fig. 3d). In contrast, EGF-maintained cultures formed KRT12-low, KRT13-positive epithelial sheets consistent with conjunctival differentiation (Figs. 3c–3e). Notably, KGF did not induce expression of the limbal basal marker SLC6A6, whose protein remained confined to a subset of basal cells even after prolonged exposure (**Extended Data** Figs. 3e, 3f), indicating that KGF promotes corneal differentiation but is insufficient to expand the limbal stem cell compartment. Clonal analysis demonstrated that individual BCAM-positive basal epithelial cells from both conjunctiva and limbus were intrinsically bipotent: KGF drove induction of KRT12 and ALDH3A1, whereas EGF maintained KRT13-positive conjunctival identity (Fig. 3f). Although *PAX6* transcript levels were comparable between conditions (**Extended Data** Fig. 3g), KGF markedly increased PAX6 protein abundance (Fig. 3g), consistent with its established role in activating corneal epithelial gene programs^28,32–34^. These findings position growth factor balance as a molecular switch governing epithelial fate at the limbal interface.

### FGFR2-MYC signaling couples lineage instruction to progenitor expansion

Suprabasal epithelial differentiation requires proliferation of basal progenitors^35,36^. In murine models, epithelial Fgfr2 deletion disrupts corneal epithelial differentiation through Pax6 dysregulation^37^. Given the enhanced stratification observed under KGF stimulation (Fig. 3e), we hypothesized that FGFR2 mediates proliferative amplification in human limbal progenitors.

**Figure 4:**
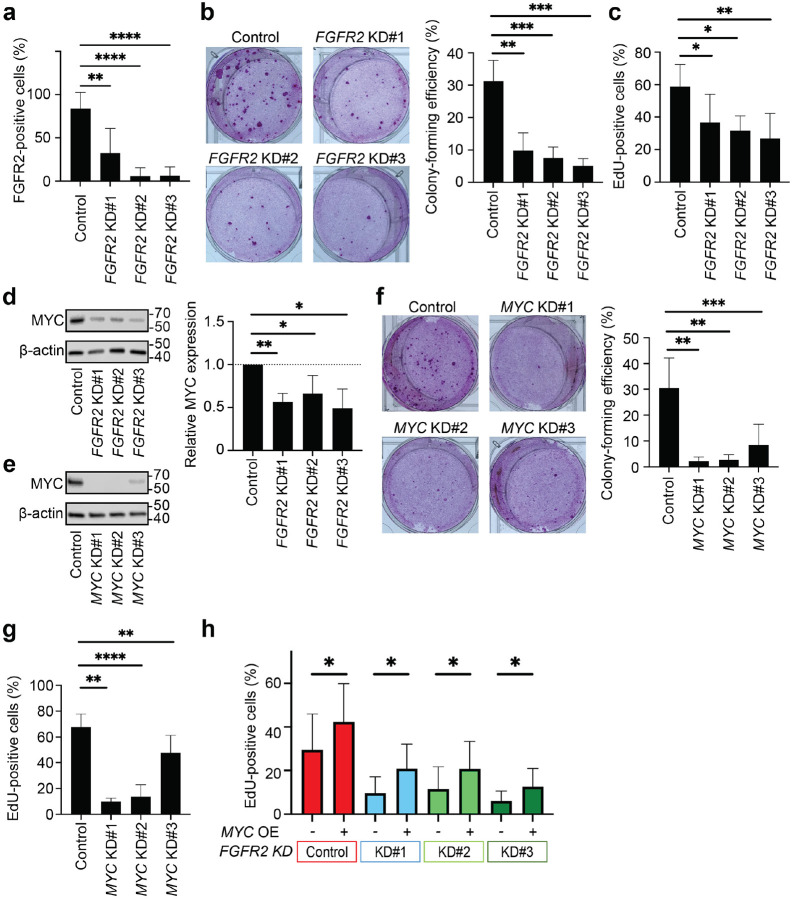
The FGFR2–MYC axis regulates proliferation of human limbal epithelial cells. **a**, Flow cytometric quantification of FGFR2-positive cells following *FGFR2* knockdown (KD) in human cultured limbal epithelial cells (mean ± SD). n = 7 donors. **p < 0.01, and ****p < 0.0001 by Dunnett’s test versus control. **b**, Left, representative images of the colonies formed by *FGFR2* KD cells compared to the control siRNA-transfected cells. Right, quantification of colony forming efficiency (CFE) (mean ± SD). n = 6 donors. **p < 0.01, and ***p < 0.001 by Dunnett’s test versus control. **c**, EdU incorporation assay demonstrating reduced proliferation following *FGFR2* KD (mean ± SD). n = 4 donors. *p < 0.05, and **p < 0.01 by Dunnett’s test versus control. **d**, Left, representative immunoblot analysis of MYC in *FGFR2* KD cells. β-actin serves as a loading control. Right, densitometric quantification of MYC protein levels. (mean ± SD). n = 4 donors. *p < 0.05, and **p < 0.01 by Dunnett’s test versus control. **e**, Representative immunoblot analysis confirming efficient *MYC* KD human limbal epithelial cells. β-actin serves as a loading control. n = 3 donors. **f**, Left, representative colony formation by *MYC* KD cells compared with control siRNA-transfected cells. Right, quantification of CFE (mean ± SD). n = 6 donors. **p < 0.01, and ***p < 0.001 by Dunnett’s test versus control. **g**, EdU incorporation assay demonstrating reduced proliferation following *MYC*KD (mean ± SD). n = 4 donors. **p < 0.01, and ****p < 0.0001 by Dunnett’s test versus control. **h**, EdU incorporation in *FGFR2* KD cells with or without MYC overexpression (OE), demonstrating partial rescue of proliferative capacity (mean ± SD). n = 4 donors. *p < 0.05 by paired t-test.

FGFR2 knockdown in primary limbal epithelial cells cultured with KGF reduced cell surface FGFR2 expression (Fig. 4a; **Extended Data** Fig. 4a). FGFR2-positive cells were markedly enriched during *in vitro* expansion (83.8 ± 18.5%, n = 7) relative to *in situ* limbus (4.5 ± 2.4%, n = 10) (Fig. 4a; **Extended Data** Fig. 4b), suggesting selective proliferative advantage under KGF exposure. FGFR2 knockdown significantly reduced colony-forming efficiency and EdU incorporation (Figs. 4b, 4c; **Extended Data** Fig. 4c), establishing an essential role for FGFR2 in limbal epithelial progenitor proliferation.

MYC, a central regulator of G1/S progression^38,39,40^, is enriched in BCAM-positive basal epithelial cells^16^. Consistent with this, *MYC* transcripts were enriched in conjunctival and limbal basal epithelial cells in CITE-seq and spatial datasets (**Extended Data** Figs. 4d, 4e). *FGFR2* knockdown reduced MYC protein expression (Fig. 4d), indicating that MYC acts downstream of FGFR2 signaling. *MYC* knockdown (Fig. 4e) phenocopied FGFR2 depletion, reducing colony formation and S-phase entry (Figs. 4f, 4g; **Extended Data** Fig. 4f), and shifting cells toward G0/G1 arrest (**Extended Data** Figs. 4g, 4h). Importantly, *MYC* overexpression partially rescued the proliferative defect induced by *FGFR2* knockdown (Fig. 4h; **Extended Data** Fig. 4i), identifying MYC as a critical effector of FGFR2-dependent progenitor expansion.

Together, these data define a KGF-FGFR2-MYC axis that links niche-derived instructive signaling to both corneal lineage specification and proliferative amplification. In this model, paracrine KGF signaling at the limbal interface selects corneal fate while simultaneously sustaining expansion of progenitor populations required for epithelial renewal.

## Discussion

Epithelial transition zones throughout the body are often viewed as sites of lineage segregation, in which discrete stem cell compartments preserve tissue identity. The human limbus has served as a canonical example of this paradigm^3,5–8^. However, our data indicate that the limbus instead functions as an instructive interface in which epithelial fate remains dynamically regulated. By resolving spatial transcriptional gradients and demonstrating single-cell bipotency in adult human tissue, we show that corneal identity is not pre-committed but actively programmed by niche-derived signals. This reframing has implications not only for ocular surface biology but also for understanding how adult epithelial identities are stabilized and remodeled at anatomical boundaries.

Spatial mapping repositions the functional conjunctival-corneal boundary to the inner edge of the limbus and reveals a sharp transition between basal transcriptional programs. Notably, limbal basal cells are transcriptionally aligned with conjunctival basal cells rather than central corneal cells, suggesting that corneal identity emerges locally under niche instruction rather than being pre-specified within a distinct progenitor pool. This organization reframes the limbus not as a static barrier but as a dynamic interface that governs epithelial fate.

Mechanistically, we identify KGF-FGFR2 signaling as an instructive axis coupling lineage specification with proliferative amplification. KGF derived from limbal fibroblasts promotes corneal differentiation through FGFR2-dependent activation of MYC and enhanced PAX6 protein abundance, whereas EGF sustains conjunctival identity. Growth factor balance thus operates as a molecular switch that programs epithelial identity at this anatomical interface. The FGFR2-MYC axis further integrates fate instruction with progenitor expansion, linking niche-derived signals to both specification and tissue renewal.

These findings provide a mechanistic framework for conjunctivalization in limbal stem cell deficiency. Rather than reflecting irreversible lineage invasion, conjunctivalization may arise from the disruption of niche-derived instructive signals that bias bipotent basal progenitors toward conjunctival fate. More broadly, this work supports a model in which adult epithelial identity at transition zones remains dynamically regulated by spatially restricted stromal cues. Such instructive niche logic provides a rationale for investigating whether similar organizing principles apply at epithelial boundaries in other organ systems.

## Methods

### Human cell cultures

The IRB reviewed the research and made the determination that it was not human subjects research. Whole globes and corneas from human donors were obtained from the Saving Sight (Kansas City, MO) and CorneaGen (Seattle, WA) eye banks. Donor characteristics are described in **Extended Data Table 3**. The conjunctival epithelium was cut from the whole globes with a 5 mm margin from the limbus. After trimming the remaining conjunctival epithelium to 2 mm from the clear cornea, limbal-corneal tissues were dissected from the posterior part of the eyes. Central corneas were punched out using an 8mm disposable biopsy punch (Integra LifeSciences, catalogue number 33–37), and the corneal endothelium was removed mechanically. After incubating these tissues (conjunctiva, limbus, and cornea) with PluriSTEM Dispase II Solution (MilliporeSigma, catalogue number SCM133) for one hour at 37°C, the epithelial cells were mechanically scraped and dissociated using TrypLE Express Enzyme (Thermo Fisher Scientific, catalogue number 12604013) for 30 min at 37°C. The harvested cells were then used for either CITE-seq or cell culture. For culture, cells were grown in DMEM/F12 medium (Thermo Fisher Scientific, catalogue number 11320033) supplemented with 10 μM Y-27632 (Tocris Bioscience, catalogue number 1254), B-27 Supplement (Thermo Fisher Scientific, catalogue number 17504044) and either 10 ng/mL KGF (Thermo Fisher Scientific, catalogue number 100 − 19) or 10 ng/mL EGF (Thermo Fisher Scientific, catalogue number 100 − 15). For sheet formation, cells dissociated with TrypLE were seeded on tissue culture inserts coated with the E8 fragment of laminin 511 at 0.5 μg/cm^2^ (iMatrix-511 SILK, Iwai North America, catalogue number N-892021), and culture media were added to both the top and bottom of the cell insert. Culture media were changed every 2–3 days.

HEK293T (Clontech Laboratories) were cultured in DMEM supplemented with 10% fetal bovine serum (Thermo Fisher Scientific) for lentivirus production.

### Immunostaining

The anterior halves of the whole globes and stratified cell sheets were dissected and processed in two ways for immunostaining: fresh-frozen blocks in Tissue-Tek Optimal Cutting Temperature (O.C.T.) compound and 10% formalin-fixation paraffin-embedded (FFPE) blocks. For the fresh-frozen samples, the tissue sections were fixed with 4% paraformaldehyde (PFA) (Electron Microscopy Sciences) for 15 min at room temperature and used for staining of SLC6A6, FGFR2, and FBLN1. For FFPE samples, the tissue sections were deparaffinized and subjected to antigen retrieval. The monolayer cultured cells were fixed with 4% PFA in chamber slides.

Tissue sections underwent permeabilization and blocking using a buffer containing 5% normal donkey serum (Jackson ImmunoResearch Laboratories) and 0.3% Triton X-100 (MilliporeSigma) for 30 min at room temperature, followed by overnight incubation with primary antibodies in the blocking buffer at 4°C. The following primary antibodies were used: mouse anti-KRT12 monoclonal antibody (mAb) (1:100, Santa Cruz Biotechnology, catalogue number sc-515882), rabbit anti-KRT12 mAb (1:400, Abcam, catalogue number ab185627), rabbit anti-BCAM polyclonal antibody (pAb) (1:100, NOVUS Biologicals, catalogue number NBP2–31994), goat anti-KRT13 pAb (1:200, Thermo Fisher Scientific, catalogue number PA5–19049), mouse anti-KRT15 mAb (1:250, Epredia, catalogue number MS1068P1), rabbit anti-NTRK2 mAb (1:250, Cell Signaling Technology, catalogue number 4607), mouse anti-SLC6A6 mAb (1:50, Santa Cruz Biotechnology, catalogue number sc-393036), rabbit anti-FGFR2 mAb (1:200, Cell Signaling Technology, catalogue number 23328), mouse anti-FBLN1 mAb (1:100, Santa Cruz Biotechnology, catalogue number sc-25281), rabbit anti-CRTAC1 pAb (1:200, Proteintech, catalogue number 13001–1-AP), and rabbit anti-MYC mAb (1:100, Abcam, catalogue number ab32072).

After rinsing with Tris-buffered saline (TBS) (Boston BioProducts), slides were incubated with Alexa Fluor 488/568/647-conjugated secondary antibodies (Abcam) for 1 hr at room temperature. Following counterstaining with Hoechst 33342 (Thermo Fisher Scientific) for 10 min at room temperature, slides were rinsed with TBS and mounted using ProLong Gold Antifade Mountant (Thermo Fisher Scientific). Images were acquired using a C2 + confocal microscope (Nikon) or ECLIPSE Ti (Nikon) and analyzed with NIS-Elements AR v4.30.01 software (Nikon).

### Flow cytometry

Cell surface expression of BCAM was detected by a previously reported method using either VioBright FITC-conjugated anti-BCAM mAb (1:11, Miltenyi Biotec, catalogue number 130-104-839) or 4 μg/mL APC-conjugated anti-BCAM mAb (1:11, Miltenyi Biotec, catalogue number 130-103-843)^15,16^. Cell surface expression of FGFR2 was detected by the previously reported method using rabbit anti-FGFR2 mAb (1:200, Cell Signaling Technology, catalogue number 23328) and anti-rabbit Alexa Fluor 488-conjugated secondary antibody (Abcam)^14^. Single-cell sorting of BCAM-positive cells was performed using a FACSDiscover S8 (BD Biosciences) for single-cell expansion. Cell analysis was performed using FACSCelesta (BD Biosciences), and data analyzed using BD FACSDiva v8.0.1 (BD Biosciences) and FlowJo v10.6.1 (BD Biosciences).

### CITE-seq

CITE-seq experiments were performed by the Center for Cellular Profiling at Brigham and Women’s Hospital. Harvested cells were blocked with Human TruStain FcX (BioLegend) for 10 min at 4°C, then incubated with TotalSeqA cell hashing antibodies (BioLegend) for 30 min at 4°C. Cells were subsequently stained with PE-conjugated anti-CD45 mAb (1:200, BioLegend, catalogue number 368510) and SYTOX Green Dead Cell Stain (1:1000, Thermo Fisher Scientific, catalogue number S34860) for 30 min at 4°C then analyzed by flow cytometry to remove hematopoietic and dead cells. Cells were next resuspended in 0.4% BSA in PBS at a concentration of 1,000 cells/μL and loaded onto a single lane (Chromium chip, 10x Genomics) for encapsulation in lipid droplets using the Single Cell 3’ kit V3.1 (10x Genomics). cDNA synthesis, gene expression and protein library generation were performed according to the 10x Genomics protocol. The generated gene expression libraries were sequenced to an average of 30,000 reads per cell, and the surface protein libraries to an average of 5,000 reads per cell, using the Illumina NovaSeq 6000 platform. ScRNA-seq reads were processed with Cell Ranger version 6.1.1., which quantified transcript counts per putative cell. Quantification was performed using the STAR aligner against the GRCh38 transcriptome (GRCh38–2020-A).

Downstream analysis was performed in the R programming environment (v4.5.0) using the settings and steps recommended by the ‘Demultiplexing with hashtag oligos (HTOs)’ vignette from Satija lab (https://satijalab.org/seurat/articles/hashing_vignette.html). Briefly, cell barcodes detected by both RNA and HTO were selected to create three separate Seurat (v5.3.0)^41^ objects, one for each donor. ‘FindVariableFeatures’ (selection.method = “mean.var.plot”) and ‘ScaleData’ were run on the Seurat objects after log normalization. HTO data were then added as an independent assay. These data were log-normalized using a centered log-ratio (CLR) transformation. ‘HTODemux’ (positive.quantile = 0.99) was used to demultiplex the cells based on HTO enrichment, after which the single cells were assigned back to their original samples. ‘FeatureScatter’ was used to confirm the mutual exclusivity of the singlets post-demultiplexing. To examine for potential batch effects, we subset the single cells before running ‘FindVariableFeatures’ (selection.method = “mean.var.plot”), ‘ScaleData’, ‘RunPCA’, ‘FindNeighbors’ (reduction = “pca”, dims = 1:10), ‘FindClusters’ (resolution = 0.4), and RunTNSE(reduction = “pca”, dims = 1:10) for t-SNE visualization. For data integration, the singlets from the three Seurat objects were combined into one Seurat object. ‘SelectIntegrationFeatures’ was used to determine features that repeatedly varied across the datasets for integration using ‘FindIntegrationAnchors’ (reduction = “rpca”) and ‘IntegrateData’. After integration, the standard workflow for visualization and clustering (‘FindClusters’ (resolution = 0.09) of single cells in a Seurat object was run. For performing differential expression analyses after integration, we selected ‘RNA’ as the ‘DefaultAssay’. Both positive and negative markers were used to assign cluster identities after running ‘FindAllMarkers’ (min.pct = 0.25, logfc.threshold = 0.25), including visualization of key markers using ‘DimPlot’, ‘VlnPlot’, and ‘FeaturePlot’. To count the number of cells assigned to each cluster (8 clusters total) from each dissected region (Conjunctiva, Limbus, and Central Cornea), we subset the cells first by donor, split them by ‘HTO_classification’, and then used ‘regionCount’. Slingshot^20^ v2.16.0 was used for trajectory analysis with cluster 4 set as the start cluster and omega = TRUE. Cells from cluster 4 (idents = “Conjunctival basal epithelial cells”) were subset for further analysis. ‘AggregateExpression’ was used to group the single cells from each hashtag type (Conjunctiva, Limbus, and Central Cornea) for each of the three donors before DESeq2 (v1.48.1) was used for pseudo-bulk analysis to compare expression between Conjunctiva and Limbus.

### Spatial transcriptomics

Formalin-fixed paraffin-embedded (FFPE) tissue sections were processed according to the Visium HD FFPE Tissue Preparation Handbook (10x Genomics). RNA quality of the FFPE tissue blocks was assessed by calculating the percentage of total RNA fragments > 200 nucleotides (DV200) from freshly collected sections. Only blocks with a DV200 ≥ 30% were used for downstream analysis. Sections were cut at 5 μm thickness and mounted within the allowable capture area on compatible blank slides as specified by the Visium CytAssist Accessory Kit (10x Genomics). Tissue sections underwent deparaffinization at 60°C for 2 hours, followed by xylene and graded ethanol washes. Hematoxylin & Eosin (H&E) staining was performed according to the protocol optimized for the Visium HD assay. For imaging, slides were loosely mounted with glycerol and a #1.5 coverslip. Following imaging by PhenoCycler Fusion (Akoya Biosciences), coverslips were removed in Milli-Q water to prepare the slides for the Visium HD workflow.

Spatial gene expression profiling was performed using the Visium HD Spatial Gene Expression platform (10x Genomics) according to the manufacturer’s protocol (CG000685 Rev D). Slides were destained in 0.1 N HCl and decrosslinked at 80°C for 30 min. The Visium Human Transcriptome Probe Set v2.0 was hybridized to tissue sections overnight at 50°C, followed by probe ligation. Captured probe products were released and transferred to a Visium HD Slide (6.5 mm Capture Area) using the Visium CytAssist instrument. Following probe release and extension, the resulting spatially barcoded ligation products were eluted.

Gene expression libraries were generated using the Dual Index Kit TS Set A. Pre-amplification material was used to determine the optimal sample index PCR cycle number via qPCR. Final libraries were purified using SPRIselect beads and quality assessed on an Agilent Fragment Analyzer 5200 with the DNF-474–1000 HS NGS Fragment Kit (1–6000 bp; protocol DNF-474–33). Libraries exhibited an average fragment size of ≈ 250 bp and were quantified using the Qubit dsDNA HS Assay Kit (Thermo Fisher Scientific). Sequencing was performed on the Singular Genomics G4 platform (Singular Genomics) using F3 100-cycle kits with paired-end 50 bp reads (Read 1: 50 cycles; Read 2: 50 cycles), targeting up to 500 million read pairs per flow cell in accordance with the manufacturer’s specifications.

Base calling and initial processing were performed using Space Ranger v4.0.1 (10x Genomics). Custom nuclei-specific binning was applied to generate cell-level expression matrices approximating single-cell resolution. Low-quality cells were excluded if they contained fewer than 20 unique molecular identifiers (UMIs), fewer than 20 detected genes, or > 20% mitochondrial transcripts. Data were normalized and variance-stabilized using SCTransform (v2 regularization) implemented in Seurat v5.4.0.

Principal component analysis (PCA) was performed on highly variable genes, and batch effects were corrected in the PCA space using Harmony v1.2.3, with sample identity specified as the batch variable. Graph-based clustering was performed using Louvain algorithm on the first 20 principal components. DGE analysis was used to identify cluster-enriched marker genes, enabling manual annotation into thirteen major cell types.

### RNA-seq

Total RNA was isolated using the mirVana miRNA Isolation Kit, with phenol (Thermo Fisher Scientific), followed by genomic DNA removal with the DNA-free DNA Removal Kit (Thermo Fisher Scientific). Libraries were prepared using Zymo-Seq RiboFree Total RNA Library Prep Kit (Zymo Research) according to the manufacturer’s instruction manual. Successful library construction was confirmed with Agilent’s D1000 ScreenTape Assay on TapeStation. RNA-seq libraries were sequenced on an Illumina NovaSeq X Plus to a sequencing depth of at least 30 million read pairs (150 bp paired-end sequencing) per sample. Trimmed reads were aligned to the reference genome using STAR v2.6.1d^42^. DEG analysis was performed using DEseq2^43^.

### Reverse transcription and quantitative PCR (qPCR)

Total RNA was reverse-transcribed into cDNA using the High-Capacity cDNA Reverse Transcription Kit (Thermo Fisher Scientific). qPCR was conducted with TaqMan Fast Universal PCR Master Mix and predesigned TaqMan Gene Expression Assay probes: *KRT12* (Hs00165015_m1), *TGFBI* (Hs00932747_m1), *ALDH3A1* (Hs00964880_m1), *CRTAC1* (Hs00907892_m1), and *GAPDH* (Hs99999905_m1) (Thermo Fisher Scientific). The cycling condition included an initial denaturation at 95°C for 20 sec, followed by 50 cycles of 95°C for 1 sec and 60°C for 20 sec using a StepOnePlus Real-Time PCR System (Thermo Fisher Scientific). Gene expression levels were normalized to *GAPDH*, which served as the endogenous control.

### Western blot

Cultured epithelial cells or sheets were lysed in RIPA buffer (Cell Signaling Technology) containing cOmplete Protease Inhibitor Cocktail (MilliporeSigma). After 30 min of ice incubation, samples were centrifuged to pellet debris, and protein concentrations were quantified using the Bio-Rad Protein Assay (Bio-Rad). Lysates were combined with SDS-sample buffer (Boston BioProducts) and 2-mercaptoethanol (MilliporeSigma), followed by denaturation at 95°C for 10 min. Proteins were run by SDS-PAGE and transferred to PVDF membranes (GE Healthcare). Membranes were blocked with 5% blotting-grade blocker (Bio-Rad) for 1 hr at room temperature, then probed overnight at 4°C with the following primary antibodies: rabbit anti-KRT12 mAb (1:5000, Abcam, catalogue number ab185627), goat anti-ALDH3A1 pAb (1:25000, GeneTex, catalogue number GTX88085), goat anti-KRT13 pAb (1:1000, Thermo Fisher Scientific, catalogue number PA5–19049), rabbit anti-MYC mAb (1:1000, Abcam, catalogue number ab32072), and rabbit anti-β-actin mAb (HRP conjugate) (1:1000, Cell Signaling Technology, catalogue number 5125). After TBS-T (Tween-20, MilliporeSigma) washes, membranes were incubated with HRP-conjugated secondary antibodies (Cell Signaling Technology) for 1 hr at room temperature. Chemiluminescent signals were generated using Western Lightning Plus-ECL (PerkinElmer) or SuperSignal^™^ West Femto Maximum Sensitivity Substrate (Thermo Fisher Scientific) and imaged on a ChemiDoc MP system (Bio-Rad) or LAS 4000 Gel Imager (Fujifilm).

### Gene knockdown and gene overexpression

Gene knockdown in the cultured cells was performed using *Silencer* Select siRNAs (Thermo Fisher Scientific) as previously described^44^. The following siRNAs were transfected by Lipofectamine RNAiMAX Transfection Reagent (Thermo Fisher Scientific): Silencer Select Negative Control No.1 siRNA, *FGFR2* siRNAs (s5173, s529187, s529188), and *MYC* siRNAs (s9129, s9130, s9131). *MYC* gene overexpression in the cultured cells was performed using lentiviruses. *MYC* lentiviral vector (Applied Biological Materials, catalogue number 312060620395) or blank lentiviral vector (Applied Biological Materials) were transfected into the HEK293T cells with 3rd Generation Packaging System Mix (Applied Biological Materials, catalogue number LV053). The collected virus-containing supernatants were concentrated by Lenti-X Concentrator (Takara Bio). 2.5 × 10^5^ IU of lentivirus and 8 μg of polybrene (Millipore Sigma, catalogue number TR-1003) were added to the 1 × 10^4^ cells, and they were cultured for 48 hrs.

### Colony-forming assay

Colony-forming assay was performed according to the established method^14,15,45^. Briefly, trypsinized limbal epithelial cells were seeded onto a mitomycin C-treated 3T3-J2 feeder cell layer at 500 live cells per well in 6-well plates and cultured for 10 days in keratinocyte culture medium (KCM) supplemented with 10 ng/mL KGF and 10 μM Y-27632. Formed colonies were fixed with 10% buffered formalin and stained with Rhodamine B (MilliporeSigma). The colony-forming efficiency was then calculated by dividing the number of colonies by 500.

#### 5-ethynyl-2’-deoxyuridine (EdU) cell proliferation assay

Click-iT EdU Alexa Fluor 488 Flow Cytometry Assay Kit (Thermo Fisher Scientific) was used for the EdU cell proliferation assay. 1 μL of EdU was added to 500 μL of cell culture media and incubated for 12 hrs. Subsequently, the cells were harvested by trypsinization, and EdU incorporation was detected according to the manufacturer’s protocol. Cell analysis was performed using FACSCelesta.

### Cell cycle analysis

The harvested cells were fixed with cold 70% ethanol and stained with FxCycle PI/RNase Staining Solution (Thermo Fisher Scientific) for 30 min at room temperature. Cell analysis was performed using FACSCelesta.

### Data and code availability

The CITE-seq and RNA-seq data were deposited to the Gene Expression Omnibus under accession numbers GSE319836 and GSE319837. The R code used for the CITE-seq is available on GitHub at git@github.com:cataalee/CITE-seq_ocular-surface.git. The spatial transcriptomics data is accessible through an interactive Shiny application at https://spatialtechnologiesunit.shinyapps.io/7ffgwc641g-5469-3b87-7ubf-8521lkk6sctvisudu3s_applet/.

### Statistical analysis

Statistical analysis was performed using GraphPad Prism 10 software. Data were presented as mean ± standard deviation (SD). Two-sided tests were employed for all statistical analyses. A paired t-test was performed to compare the two groups, and Dunnett’s test was performed to compare the groups with more than two categories. Specific statistical methods used were depicted in the figure legends. Immunostaining and Western blot images are representative of at least three independent repetitions. The significance level was set at p < 0.05. *p < 0.05, **p < 0.01, ***p < 0.001, and ****p < 0.0001.

## Supplementary Material

Supplementary Files

This is a list of supplementary files associated with this preprint. Click to download.

- ExtendedData.docx

Supplementary information is available for this paper.
